# Neuroprotective effect of a novel gastrodin derivative against ischemic brain injury: involvement of peroxiredoxin and TLR4 signaling inhibition

**DOI:** 10.18632/oncotarget.18773

**Published:** 2017-06-28

**Authors:** Xiao-Na Mao, Hong-Jing Zhou, Xiao-Jia Yang, Li-Xue Zhao, Xi Kuang, Chu Chen, Dong-Ling Liu, Jun-Rong Du

**Affiliations:** ^1^ Department of Pharmacology, Key Laboratory of Drug Targeting and Drug Delivery Systems, West China School of Pharmacy, Sichuan University, Chengdu, 610041, China; ^2^ Sichuan Academy of Chinese Medicine Sciences, Chengdu, 610041, China

**Keywords:** ischemic stroke, neuroinflammation, peroxiredoxins, therapeutic window, gastrodin derivative

## Abstract

The inhibition of extracellular inflammatory peroxiredoxin (Prx) signaling appears to be a potential therapeutic strategy for neuroinflammatory injury after acute ischemic stroke. Gastrodin (Gas) is a phenolic glycoside that is used for the treatment of cerebral ischemia, accompanied by regulation of the autoimmune inflammatory response. The present study investigated the neuroprotective effects of Gas and its derivative, Gas-D, with a focus on the potential mechanism associated with inflammatory Prx–Toll-like receptor 4 (TLR4) signaling. Gas-D significantly inhibited Prx1-, Prx2-, and Prx4-induced inflammatory responses in RAW264.7 macrophages and H2O2-mediated oxidative injury in SH-SY5Y nerve cells. In rats, intraperitoneal Gas-D administration 10 h after reperfusion following 2-h middle cerebral artery occlusion (MCAO) ameliorated neurological deficits, brain infarction, and neuropathological alterations, including neuron loss, astrocyte and microglia/macrophage activation, T-lymphocyte invasion, and lipid peroxidation. Delayed Gas-D treatment significantly inhibited postischemic Prx1/2/4 expression and spillage, TLR4 signaling activation, and inflammatory mediator production. In contrast, Gas had no significant effects in either cell model or in MCAO rats under the same conditions. These results indicate that Gas-D may be a drug candidate with an extended therapeutic time window that blocks inflammatory responses and attenuates the expression and secretome of inflammatory Prxs in acute ischemic stroke.

## INTRODUCTION

Ischemic stroke is a leading cause of morbidity and mortality worldwide, with severe socioeconomic consequences and poor quality of life among recovered patients because of cognitive disability [1]. The recanalization of occluded cerebral blood vessels and thrombolytic therapy are the currently available therapeutic approaches for acute ischemic stroke [2]. However, reperfusion therapy has achieved limited clinical success because of the narrow therapeutic time window and secondary brain injury, such as blood-brain barrier disruption, hemorrhagic transformation, and massive brain edema [3]. There is an unmet need for safe and effective therapies with an extended therapeutic time window for the treatment of ischemic stroke.

Numerous studies have shown that immunoinflammation is an essential event in the progression of ischemic brain injury after ischemic stroke [4–7]. Recent evidence suggests that danger-associated molecular patterns (DAMPs) that are released from ischemic brain cells may function as endogenous ligands of Toll-like receptors (TLRs) and induce the activation of resident glia and infiltration of blood-derived immune cells, resulting in a postischemic immunoinflammatory response and brain damage [8]. To date, more than 10 types of DAMPs have been identified in the ischemic brain. Among these, peroxiredoxins (Prxs) merit special attention because they are more highly expressed in the brain than in other tissues. Moreover, Prxs may be further upregulated by an increase in oxidative stress after ischemic brain injury and are extracellularly released over 12 h after stroke onset, which may provide a wide therapeutic time window for brain ischemia [8–11]. Three isoforms of Prxs (Prx1, Prx2, and Prx4) directly induced an inflammatory response in cultured macrophages by activating TLR4 signaling and producing inflammatory mediators [11–13]. Therefore, novel neuroprotectants that may directly block inflammatory Prxs-TLR4 signaling or reduce the redox-dependent expression and spillage of inflammatory Prxs after cerebral ischemia may be a promising antineuroinflammatory approach with a therapeutic time window of more than 12 h for acute ischemic stroke.

**Figure 1:**
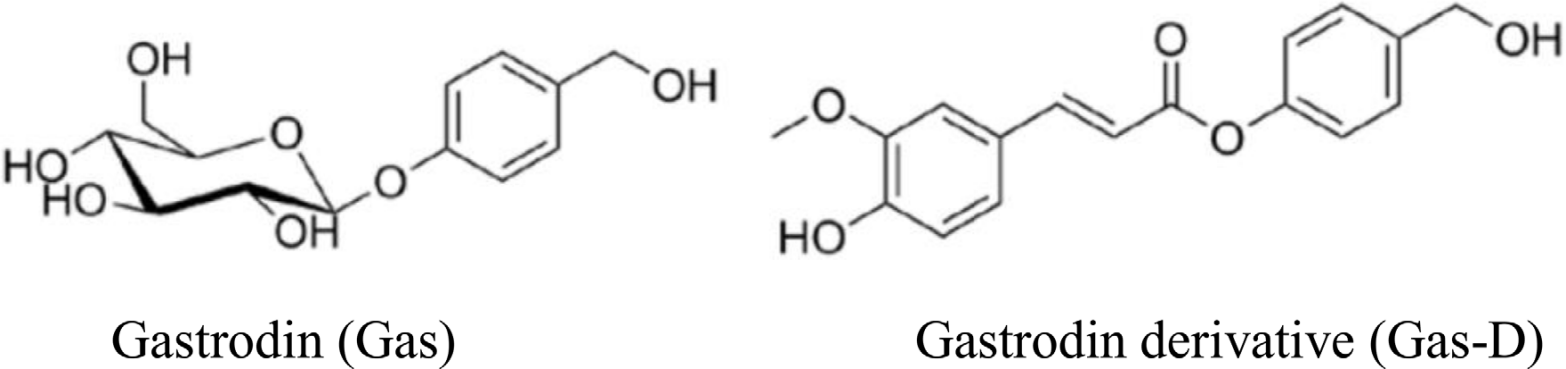
Chemical structures of gastrodin (Gas) and its derivative (Gas-D)

Gastrodin (Gas) is the most active constituent from the medicinal plant Gastrodiae Rhizoma, also known as Tianma in Chinese [14]. To date, multiple preparations of Gas have been applied clinically for the treatment of various neurovascular disorders, such as angioneurotic headache and migraine [15]. Pharmacological studies have shown that an intraperitoneal (i.p.) injection of 100 mg/kg Gas at the start of reperfusion after 2-h middle cerebral artery occlusion (MCAO) in rats or after 1-h MCAO in mice significantly reduced terminal deoxynucleotidyl transferase-mediated dUTP nick end labeling (TUNEL)-, S100β-, and inducible nitric oxide synthase (iNOS)-positive cells in the ischemic boundary zone in MCAO rats [16] and attenuated cerebral ischemic damage, accompanied by the upregulation of antioxidant enzymes (superoxide dismutase, HO-1, and Nrf2), downregulation of activation of the mitochondria-dependent caspase 3 apoptosis pathway, and downregulation of the production of inflammatory mediators (e.g., tumor necrosis factor a [TNFα] and interleukin-1b [IL-1β]) [17]. Oral administration of Gas sustained-release tablets, 150 mg twice daily for 28 days, significantly increased the frequencies of regulatory T cells (Treg) and decreased IL-17 levels in peripheral blood in patients with a high risk of transient ischemic attack in posterior circulation compared with control patients, suggesting the potential inhibitory effect of Gas on the autoimmune inflammatory response in cerebral ischemia [18]. However, Gas is a phenolic glycoside with a simple chemical structure (Figure 1) and low permeability of the blood-brain barrier (BBB). It reaches relatively low levels and has weak efficacy in the central nervous system (CNS) [19]. To develop a safer and more effective agent for the treatment of ischemic stroke, we synthesized a series of Gas derivatives, one of which was a novel Gas ester derivative (Gas-D; Figure 1). Gas-D consists of the main moieties of both Gas and ferulic acid (Supplementary Figure 1), which may give Gas-D a more prominent CNS profile [20]. Ferulic acid is one of the main effective components of various medicinal plants, such as Radix Angelicae sinensis [21]. Ferulic acid is another commonly used phenolic acid for the treatment of cardio- and cerebrovascular diseases [22]. The pharmacodynamics of ferulate have been shown to be highly associated with its direct clearance of reactive oxygen species (ROS) and antioxidant activity [23]. The present study investigated the effects of Gas and Gas-D on inflammatory and oxidative responses and cerebral ischemia, with a specific focus on the potential mechanism associated with postischemic inflammatory Prx1/2/4-TLR4 signaling. We first evaluated the effects of Gas and Gas-D on exogenous recombinant Prx1/2/4-induced inflammatory responses in RAW264.7 macrophages and H_2_O_2_-induced oxidative injury in SH-SY5Y nerve cells. We also performed comparative pharmacodynamic studies of the effects of delayed Gas and Gas-D treatment (100 mg/kg, i.p.) 10 h after reperfusion following 2-h MCAO to evaluate postischemic neurological outcomes, neuropathological changes, redox-dependent Prx expression, extracellularly released Prx-TLR4 signaling, and immunoinflammatory responses in the brain. The results showed that Gas-D is a promising drug candidate with a wide therapeutic time window and greater therapeutic efficacy than Gas for acute ischemic stroke.

## RESULTS

### Effects of Gas and Gas-D on Prx-induced inflammatory response in macrophages

**Figure 2:**
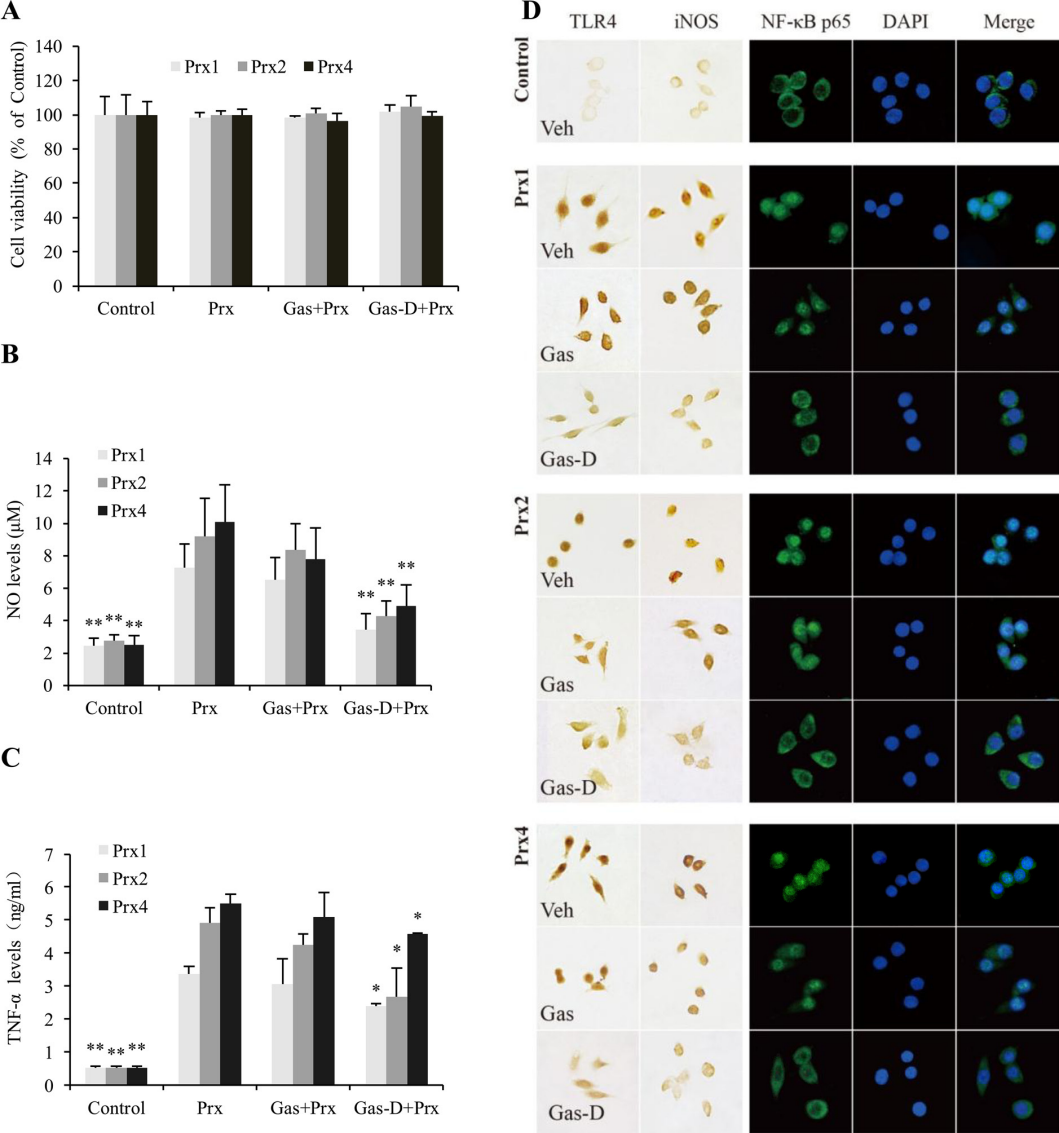
Effects of gastrodin (Gas) and its derivative (Gas-D) on the peroxiredoxin (Prx)-induced inflammatory response in macrophages RAW264.7 cells were pretreated for 1 h with vehicle, 20 μM Gas, or 20 μM Gas-D and then incubated with vehicle (Control) or the indicated Prx (Prx1, Prx2, or Prx4) at a dose of 20 nM for 24 h. (**A**) Cell viability was examined by the MTT assay. The results are expressed as a percentage of the control group. (**B**) Nitric oxide levels in the culture medium were measured using a NO kit. (**C**) TNF-α content in the culture medium was measured using ELISA. The data are representative of results that were obtained from three independent experiments with six replicates. ^*^p < 0.05, ^**^p < 0.01, vs. corresponding Prx-treated group. (**D**) Immunostaining images of TLR4, iNOS, and NF-κB p65 are representative of three independent experiments. Cell nuclei were stained using 4, 6-diamidino-2-phenylindole (DAPI).

The potential cytotoxicity of Gas and Gas-D in 20 nM Prx1-, Prx2-, and Prx4-stimulated RAW264.7 macrophages was first examined by the MTT test. The MTT results showed that both Gas and Gas-D (5–20 μM) had no significant cytotoxicity in each subtype of Prx-stimulated cells (Figure 2A, Supplementary Figure 2).

As shown in Figure 2B–2D, 20 nM Prx1, Prx2, and Prx4 significantly induced the production of inflammatory mediators, reflected by increases in NO and TNF-α levels and iNOS immunoreactivity. Pretreatment with 20 μM Gas-D for 1 h significantly decreased Prx-induced NO and TNF-α production and iNOS expression compared with the corresponding Prx-alone groups (*p* < 0.05 or 0.01). Gas had no apparent effect on the production of inflammatory mediators under the same conditions. To further explore the potential mechanism by which Gas-D inhibited the Prx-stimulated inflammatory response in macrophages, immunostaining was applied to detect activation of the TLR4/NF-κB signaling pathway. All three Prx subtypes strongly enhanced the immunoreactivity of TLR4 and nuclear translocation of NF-κB p65 that was predominantly located in the cellular cytoplasm in the control group (Figure 2D). Notably, 10 μM Gas-D significantly reduced TLR4 expression and NF-κB p65 nuclear translocation compared with the corresponding Prx-treated group, suggesting that the inhibitory effect of Gas-D against Prx-induced inflammation may be associated with downregulation of the activation of TLR4/NF-κB signaling in macrophages. In contrast, Gas did not significantly affect TLR4 expression or NF-κB activation compared with the corresponding Prx-alone treatment.

### Effects of Gas and Gas-D on H_2_O_2_-induced cell injury in nerve cells

To explore the effects of Gas and Gas-D on H_2_O_2_-induced cell injury, SH-SY5Y cells were treated with Gas, Gas-D, and H_2_O_2_ at the indicated concentrations for 24 h. The MTT assay showed that both Gas and Gas-D (1–10 μM) had no significant cytotoxicity, whereas H_2_O_2_ (200–400 μM) concentration-dependently induced cytotoxicity (Supplementary Figure 3). Therefore, 300 μM H_2_O_2_, which resulted in cell viability of 61.4% ± 4.8% relative to the control group, was used for the subsequent experiments.

**Figure 3:**
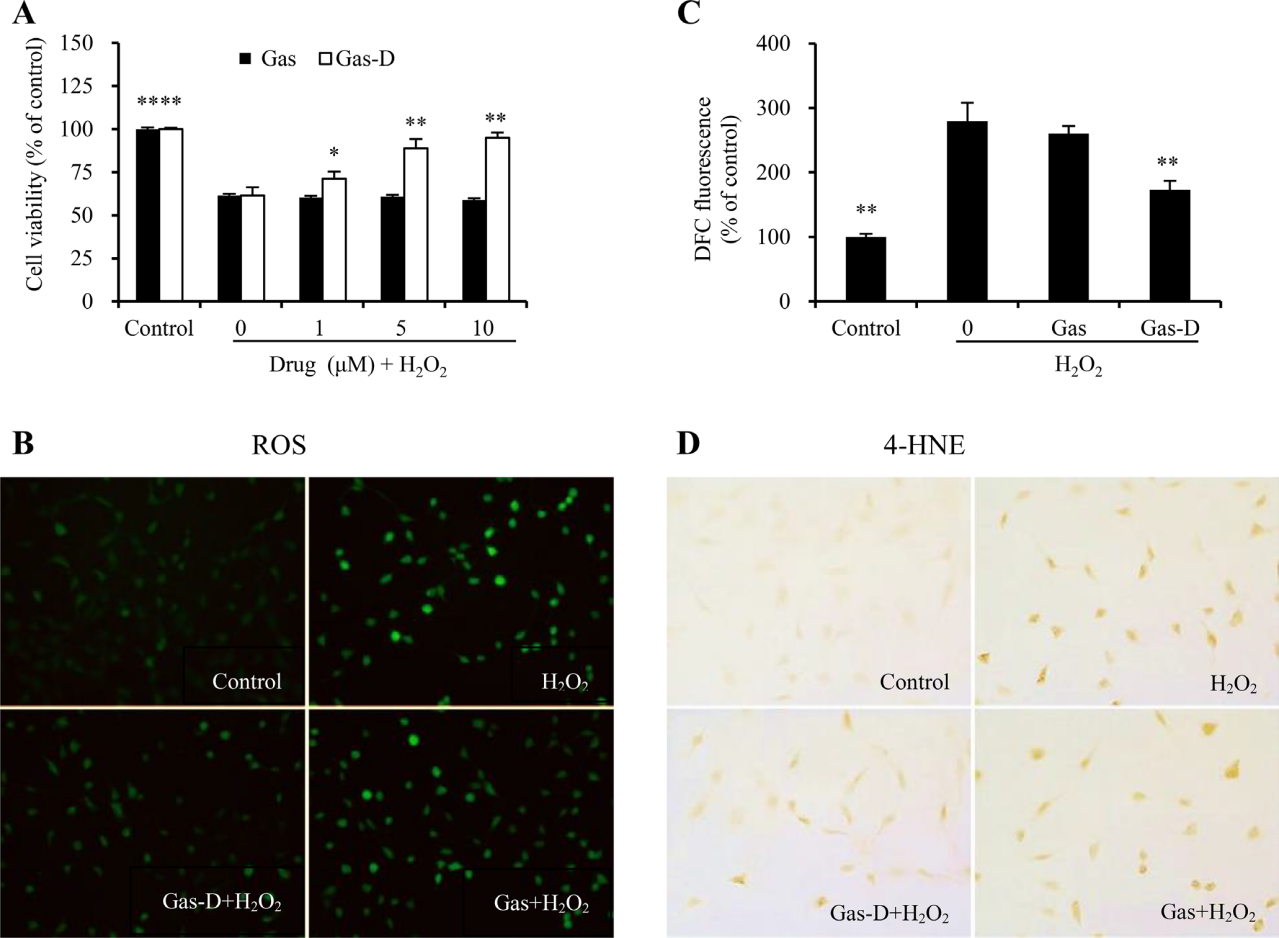
Effects of gastrodin (Gas) and its derivative (Gas-D) on H_2_O_2_-induced oxidative injury in nerve cells (**A**) Effects of Gas and Gas-D on H_2_O_2_-induced cytotoxicity. Cells were incubated with Gas or Gas-D at the indicated concentrations for 24 h and then 300 μM H_2_O_2_ with additional incubation for 24 h. Cell viability was examined by the MTT assay. (**B**) Effects of Gas and Gas-D on H_2_O_2_-induced ROS production in cells. After pretreatment with 10 μM Gas or Gas-D for 24 h, the cells were exposed to 300 μM H_2_O_2_ for 1 h. Intracellular ROS levels were examined by the ROS assay kit. (**C**) The quantitative analysis of ROS was performed by measuring the mean fluorescent intensity of three random fields (20´ objective). (**D**) Effects of Gas and Gas-D on H_2_O_2_-induced lipid peroxidation. Photomicrographs are representative of three independent experiments. The results are representative of three independent experiments. The data are expressed as mean ± SEM (*n* = 6). ^*^p < 0.05, ^**^p < 0.01, vs. H_2_O_2_ alone.

As shown in Figure 3A, pretreatment with Gas-D (1–10 μM) for 24 h dose-dependently attenuated H_2_O_2_-induced cell injury (*p* < 0.05 or 0.01, vs. H_2_O_2_ group), whereas Gas had no protective effect against H_2_O_2_-induced cell injury under the same conditions. To further investigate the effects of Gas and Gas-D on H_2_O_2_-induced oxidative injury, the production of intracellular ROS and expression of 4-HNE (a marker of lipid peroxidation) were examined. Compared with vehicle-treated controls, H_2_O_2_ significantly increased ROS generation and 4-HNE immunoreactivity, which were decreased by 10 μM Gas-D (Figure 3B–3D). In contrast, Gas did not significantly affect the levels of ROS or 4-HNE compared with H_2_O_2_-alone treatment.

### Effects of Gas and Gas-D on cerebral ischemia outcomes

**Figure 4:**
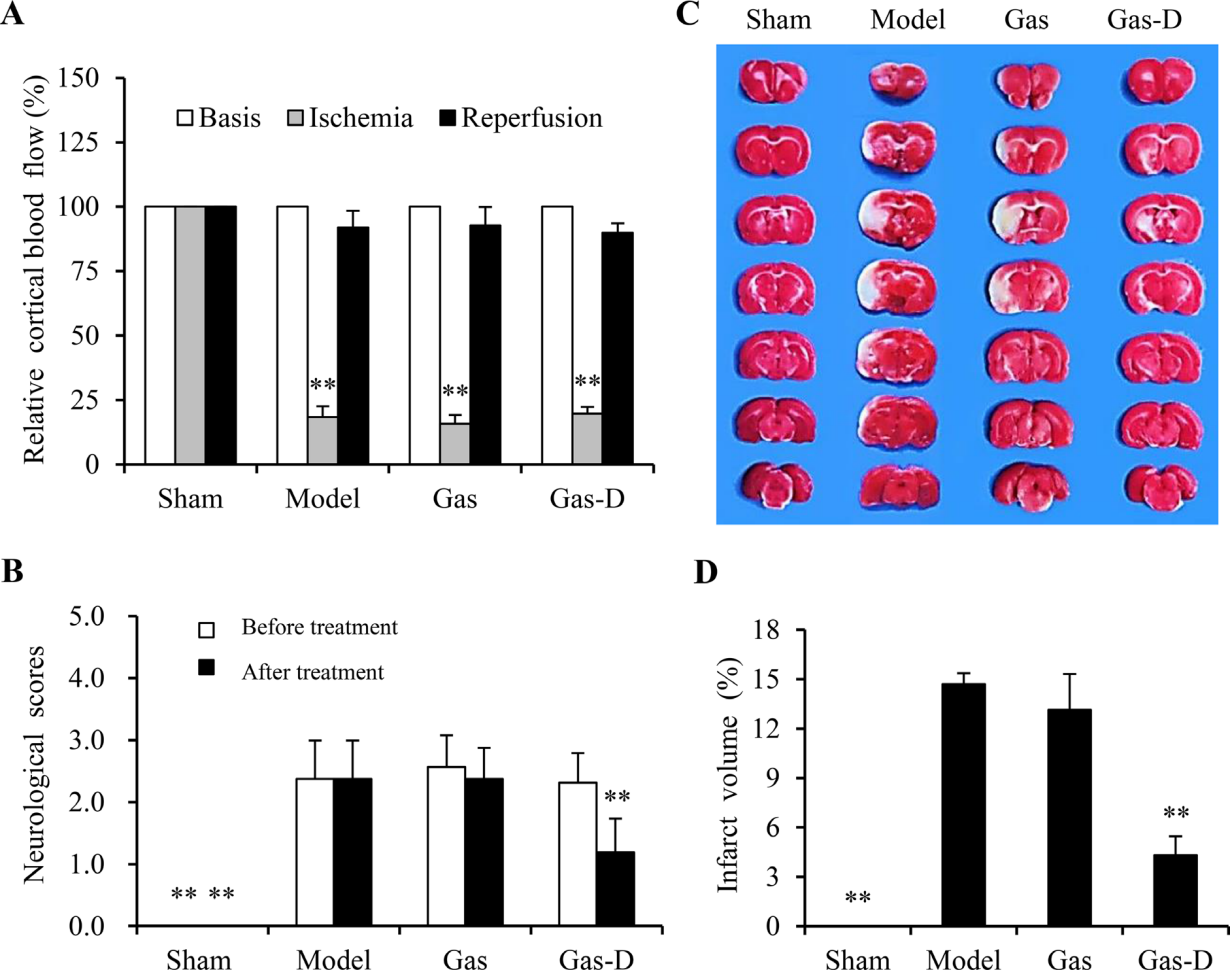
Effects of gastrodin (Gas) and its derivative (Gas-D) on neurobehavioral deficits and brain injury in transient MCAO rats Ten hours after reperfusion following 2-h MCAO, vehicle (Sham and Model), 100 mg/kg Gas, or 100 mg/kg Gas-D was administered intraperitoneally. (**A**) In MCAO rats, cortical blood flow (CBF) decreased by more than 80% after MCAO and was restored by up to 80% after reperfusion as measured by laser Doppler flowmetry. (**B**) Neurobehavioral deficits were evaluated 10 and 22 h after reperfusion following MCAO (before and after treatment). (**C**) Representative photographs of TTC-stained coronal brain sections that show viable (red) and dead (white) tissue 22 h after reperfusion following MCAO. (**D**) Cerebral infarct volume expressed as a percentage of whole brain volume. The data are expressed as mean ± SEM (*n* = 16 per group, except 6 in the TTC staining group). ^**^p < 0.01, compared with vehicle-treated model group.

To explore the effects of Gas and Gas-D on ischemic brain injury, male rats were subjected to 2 h MCAO followed by 22 h reperfusion, and CBF was monitored by laser Doppler flowmetry. MCAO rats were randomly divided into three groups according to similar ischemia and reperfusion (∼80% decrease in CBF after occlusion and > 80% recovery of CBF after reperfusion; Figure 4A) and neurobehavioral deficits (Figure 4B), followed by intraperitoneal administration of vehicle, 100 mg/kg Gas, or 100 mg/kg Gas-D 10 h after reperfusion following MCAO. As shown in Figure 4B–4D, delayed treatment with Gas-D effectively attenuated neurological deficits and infarct volume (*p* < 0.01, vs. vehicle-treated MCAO group). Gas treatment did not improve neurological outcomes compared with vehicle-treated MCAO controls. These data demonstrate the potent neuroprotective effects of Gas-D against ischemic brain injury within a therapeutic time window > 10 h after the onset of focal cerebral ischemia.

### Effects of Gas and Gas-D on neuropathological changes induced by cerebral ischemia

**Figure 5:**
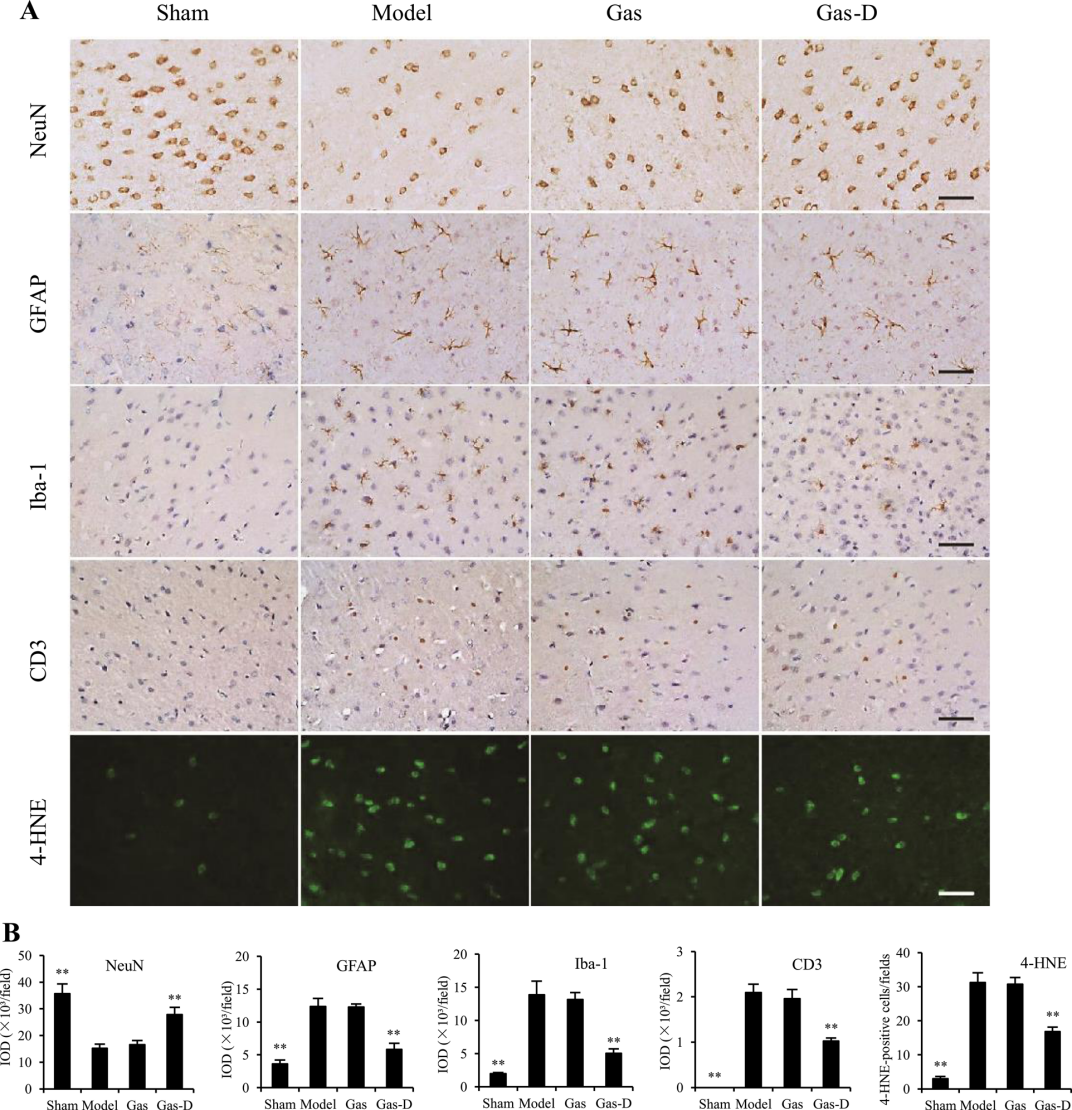
Effects of gastrodin (Gas) and its derivative (Gas-D) on neuropathological alterations in transient MCAO rats Ten hours after reperfusion following 2-h MCAO, vehicle (Sham and Model), 100 mg/kg Gas, or 100 mg/kg Gas-D was administered intraperitoneally. Ischemic brain tissues were processed for immunohistochemistry 22 h after reperfusion following MCAO. (**A**) Representative photomicrographs of NeuN-immunopositive neurons, GFAP-positive astrocytes, Iba-1-positive microglia/macrophages, and CD3-positive T lymphocytes, and 4-HNE immunoreactivity. (**B**) Quantitative image analysis was performed based on the integrated optical density (IOD) of positive immunostaining (NeuN and GFAP, Iba-1 and CD3) or the number of 4-HNE-positive cells in the ischemic cortex. Scale bar = 50 μm. The data are expressed as mean ± SEM (*n* = 6 per group). **p < 0.01, compared with vehicle-treated model group.

Twenty-two hours after reperfusion following 2-h MCAO, pathological alterations in the brain were examined immunohistochemically. Figure 5A shows that sham-operated rats had a large number of NeuN-immunopositive neurons, which were greatly decreased in the ischemic cortex in the vehicle-treated MCAO group (*p* < 0.01). Consistent with the ischemic stroke outcomes (Figure 4), delayed treatment with Gas-D significantly restored neuronal cells in the ischemic cerebral cortex compared with the MCAO group (*p* < 0.01), whereas Gas had no effect on neuronal damage after cerebral ischemia.

Numerous studies have shown that both the neuroinflammatory response and oxidative stress are involved in postischemic brain injury. Consistent with previous reports [29, 31], the present results showed that MCAO induced the activation of astrocytes and microglia and infiltration of blood-derived macrophages and T lymphocytes, reflected by increases in the immunoreactivity of GFAP, Iba-1, and CD3 in the ischemic cerebral cortex compared with the sham group (*p* < 0.01; Figure 5B). MCAO increased the immunoreactivity of 4-HNE (a late-phase marker of lipid peroxidation) compared with sham controls (*p* < 0.01; Figure 5B). Notably, Gas-D reduced the expression of all biomarkers of immunoinflammatory cells and 4-HNE compared with the MCAO group (*p* < 0.01), whereas Gas did not affect these neuropathological alterations after cerebral ischemia (*p* > 0.05, vs. MCAO group). Altogether, the present results suggest that Gas-D exerted a potent protective effect against MCAO-induced neuropathological changes, including decreases in neuronal loss, glial activation, blood-derived immune cell infiltration, and oxidative injury.

### Effects of Gas and Gas-D on the expression and spillage of Prxs in the ischemic brain

**Figure 6:**
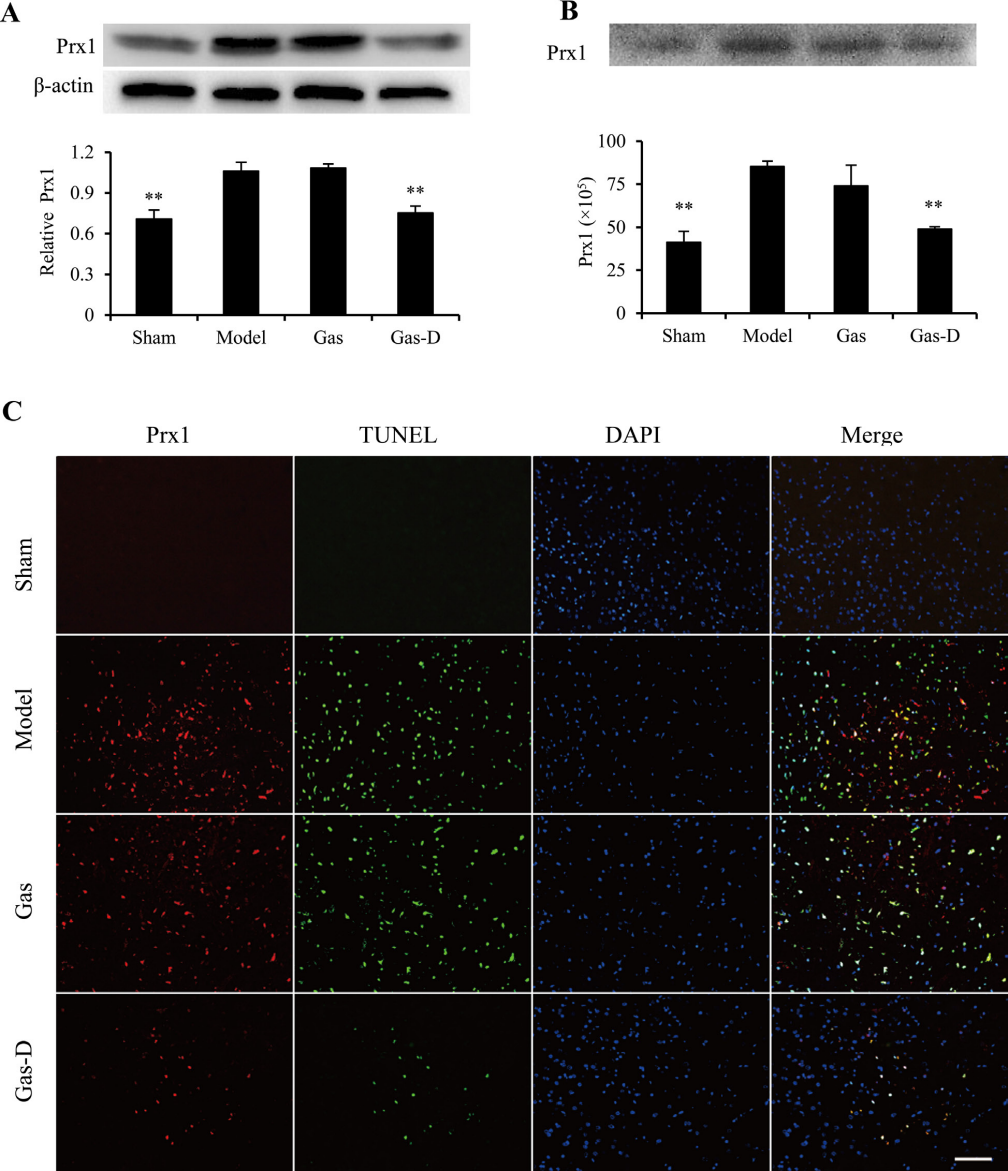
Effects of gastrodin (Gas) and its derivative (Gas-D) on the expression and spillage of peroxiredoxin 1 (Prx1) in transient MCAO rats Ten hours after reperfusion following 2-h MCAO, vehicle (Sham and Model), 100 mg/kg Gas, or 100 mg/kg Gas-D was administered intraperitoneally. The ischemic cortex in the ipsilateral hemisphere or cerebrospinal fluid (CSF) was collected for Western blot or immunohistochemistry 22 h after reperfusion following MCAO. (**A**) Representative Western blots of Prx1 in the ischemic brain. Prx1 expression is expressed as a ratio relative to β-actin. (**B**) Representative immunoblots of Prx1 in CSF. All of the lanes were loaded with 20 μl of CSF. Band intensity is expressed in arbitrary units after subtracting the background signal of each band. (**C**) Double staining using Prx1 (red) and TUNEL (green) immunohistochemistry in the ischemic cortex. Nuclear counterstaining was performed using DAPI (blue). Merged photographs present Prx1 localization in ischemic brain cells. Scale bar = 100 μm. The data are expressed as mean ± SEM (*n = 3* per group). ^**^p < 0.01, compared with vehicle-treated model group.

**Figure 7:**
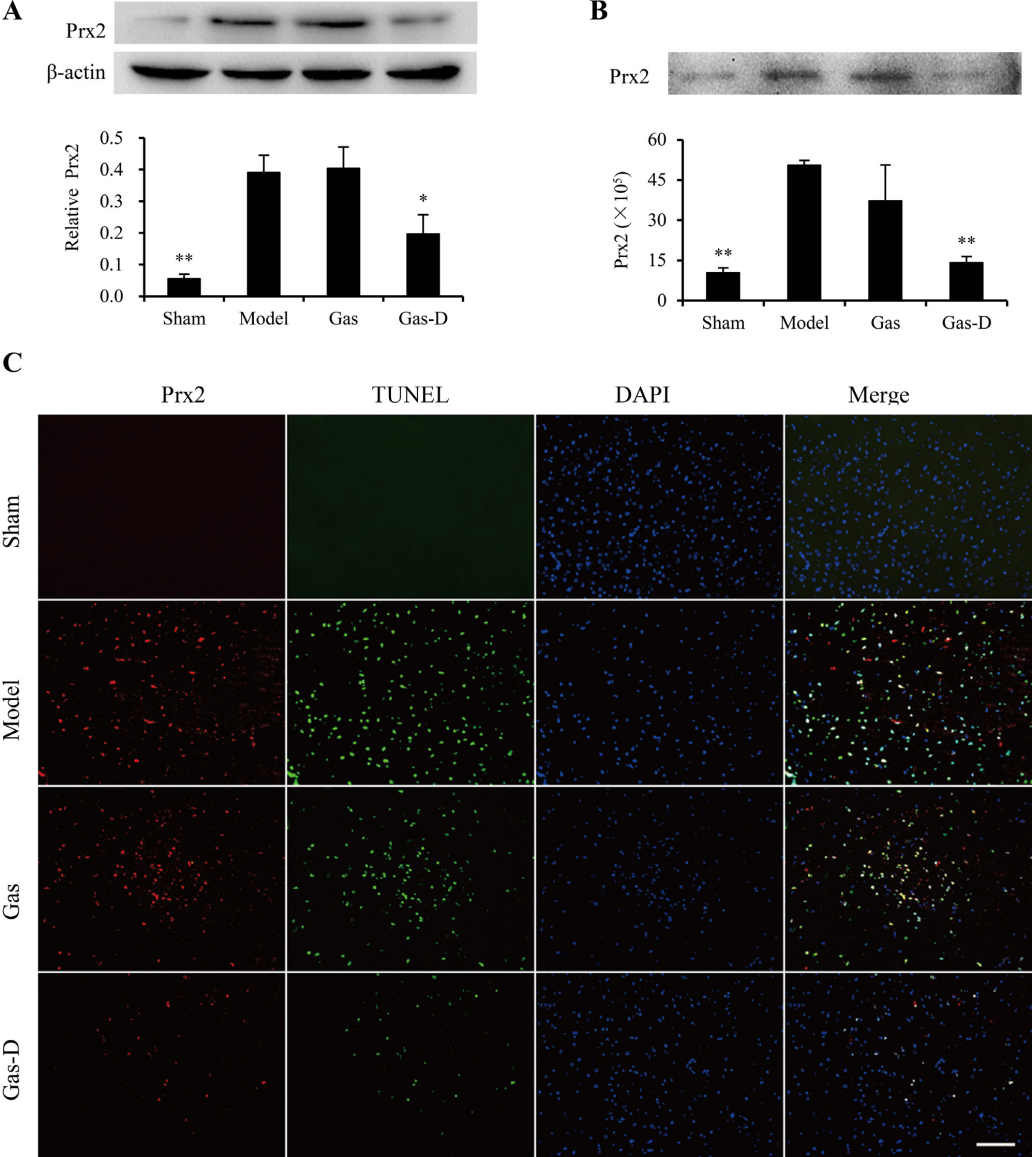
Effects of gastrodin (Gas) and its derivative (Gas-D) on the expression and spillage of peroxiredoxin 2 (Prx2) in transient MCAO rats ^*^p < 0.05, compared with vehicle-treated model group. See Figure 6 for details.

**Figure 8:**
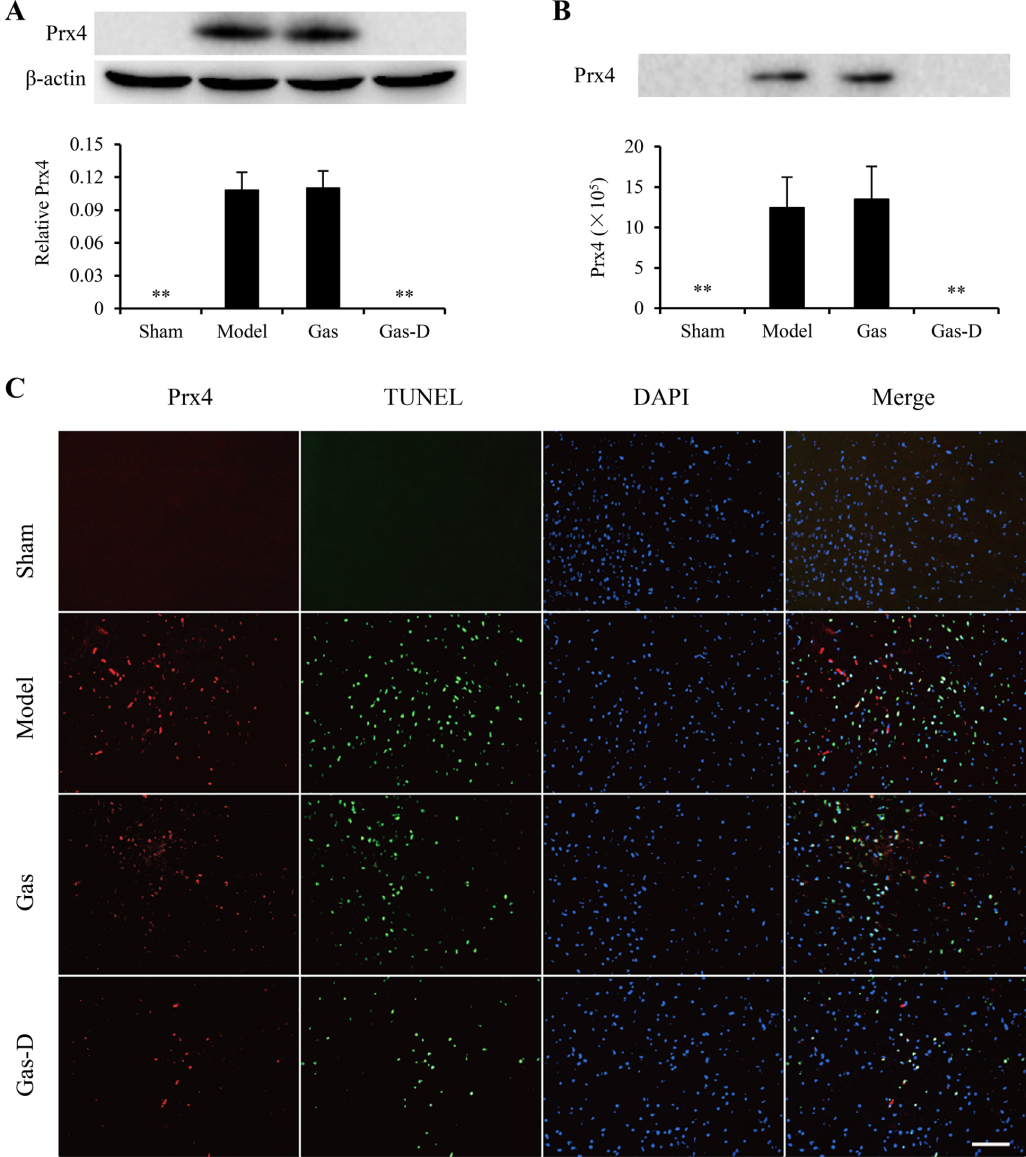
Effects of gastrodin (Gas) and its derivative (Gas-D) on the expression and spillage of peroxiredoxin 4 (Prx4) in transient MCAO rats See Figure 6 for details.

Our previous study showed that only three isoforms of exogenous Prxs (Prx1, Prx2, and Prx4) are inflammatory and may trigger an inflammatory response through the induction of TLR4/NF-kB signaling and subsequent production of proinflammatory mediators in macrophages, suggesting that these Prx subtypes likely act as extracellular DAMPs that aggravate the progression of neuroinflammatory injury in the ischemic brain [13]. In the present study, we examined the expression and spillage of Prx1, Prx2, and Prx4 in the ischemic brain by immunoblotting and immunohistochemistry. As shown in Figure 6–8, the levels of expression of Prx1, Prx2, and Prx4 in the ischemic cortex markedly increased in the MCAO group compared with sham-operated controls (*p* < 0.01). Immunoblotting indicated that the levels of Prx1, Prx2, and Prx4 in CSF significantly increased in the MCAO group compared with the sham group (*p* < 0.01; Figure 6B, 7B, 8B). Immunofluorescent staining indicated that the immunoreactivity of Prx1, Prx2, and Prx4 was mainly observed around TUNEL-positive cells in the ischemic cortex in the MCAO group, accompanied by higher expression in the extracellular compartment of dead or dying cells (Figure 6C, 7C, 8C). Interestingly, delayed treatment with 100 mg/kg Gas-D 10 h after reperfusion following MCAO significantly decreased the postischemic expression and extracellular release of Prx1, Prx2, and Prx4 (*p* < 0.05 or 0.01). In contrast, no difference was observed in the expression or spillage of Prx1, Prx2, and Prx4 in MCAO rats that received vehicle or Gas treatment (*p* > 0.05). These data suggest that the neuroprotective effect of Gas-D is at least partially associated with the downregulation of inflammatory Prx expression and extracellular release, thereby leading to a decrease in endogenous TLR ligands and subsequent inhibition of TLR signaling pathways in focal cerebral ischemia.

### Effects of Gas and Gas-D on brain ischemia-induced activation of TLR4 signaling and downstream response genes

**Figure 9:**
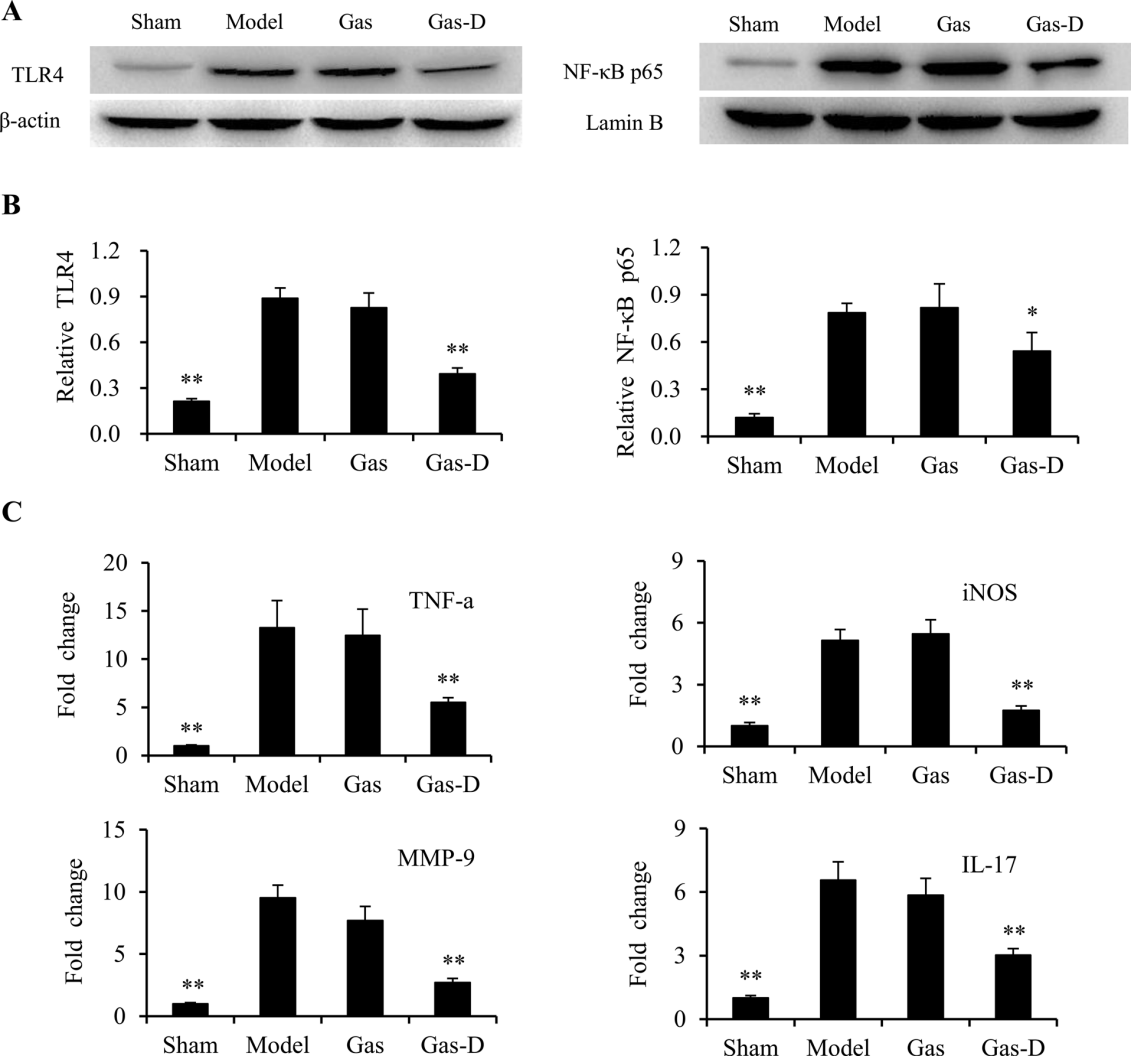
Effects of gastrodin (Gas) and its derivative (Gas-D) on TLR4/NF-kB signaling and inflammatory response in transient MCAO rats Ten hours after reperfusion following 2-h MCAO, vehicle (Sham and Model), 100 mg/kg Gas, or 100 mg/kg Gas-D was administered intraperitoneally. The ischemic cortex in the ipsilateral hemisphere was collected for Western blot and qPCR 22 h after MCAO. (**A, B**) Representative Western blots and quantitative analysis of TLR4 expression and nuclear translocation of NF-κB p65. The ratios of TLR4 to β-actin and NF-κB p65 to Lamin B were analyzed densitometrically. (**C**) mRNA expression levels of inflammatory mediators (TNF-a, iNOS, MMP-9, and IL-17) determined by qPCR. The results were first normalized to the corresponding reporter gene β-actin and are presented as fold changes relative to the sham-operated group. The data are expressed as mean ± SEM (*n =* 3–4 per group). ^*^p < 0.05, ^**^p < 0.01, compared with vehicle-treated model group.

To further elucidate the mechanism that underlies the neuroprotective effects of Gas-D against postischemic brain injury, we further compared the effects of Gas and Gas-D on the TLR4 signaling pathway and downstream expression of inflammatory mediators in the transient MCAO rat brain using Western blot and qPCR. As shown in Figure 9A and 9B, cerebral ischemia significantly activated the TLR4/NF-kB signaling pathway, reflected by increases in the expression of TLR4 and nuclear translocation of NF-kB p65 in the MCAO group compared with sham controls (*p* < 0.01). Delayed treatment with 100 mg/kg Gas-D but not Gas significantly inhibited TLR4 expression and NF-kB activation in ischemic cerebral tissue compared with the vehicle-treated MCAO group (*p* < 0.05 and 0.01; Figure 9). Consistent with postischemic TLR4/NF-kB signaling activation, increases were observed in the mRNA levels of the proinflammatory mediators TNF-a, iNOS, matrix metalloproteinase-9 (MMP-9), and IL-17 in the ischemic brain (*p* < 0.01, vs. sham controls), which were significantly reduced by Gas-D (*p* < 0.01, vs. MCAO group). Gas had no effect on the production of proinflammatory mediators. These results suggest that Gas-D decreased the production of proinflammatory mediators from both activated parenchymal glia and infiltrating blood-derived immune cells, such as T lymphocytes, at least partially by inhibiting inflammatory Prx-TLR4 signaling activation in the ischemic brain.

## DISCUSSION

The newly synthesized compound Gas-D is a potential neuroprotective agent that has a patent pending [20]. The present study found that Gas-D significantly prevented the Prx1/2/4-induced inflammatory response in RAW264.7 macrophages and H_2_O_2_-induced oxidative injury in SH-SY5Y nerve cells. Delayed treatment with Gas-D 10 h after reperfusion following 2-h MCAO effectively improved neurological outcomes after acute ischemic stroke in rats, accompanied by decreases in the immunoinflammatory response, oxidative stress, Prx1/2/4 expression and spillage, and TLR4 signaling activation. In contrast, Gas had no effect in either the cell models or in the rat MCAO model under the same conditions. These findings show that the inhibition of Prx1/2/4-TLR4 signaling and neuroinflammation by the small-molecule Gas-D may be an innovative therapeutic strategy with a wide therapeutic time window for the treatment of acute ischemic stroke. Novel neuroprotective agents that target crucial pathophysiological events in postischemic neuroinflammatory injury have long been regarded as potential therapeutic strategies for acute ischemic stroke [5, 24, 25]. Despite intensive studies in the past 20 years, the pathogenesis of neuroinflammation after ischemic stroke has not been fully clarified, thus causing slow progress in the development of effective antiinflammatory neuroprotectants [26]. Numerous studies have suggested that both oxidative stress and neuroinflammation are implicated in the pathogenesis of ischemic stroke, but the underlying mechanisms have remained largely unclear. *One notable* advance in elucidating the underlying mechanisms was identification of the potential role of a unique type of DAMPs (i.e., the Prx family proteins) in oxidative and inflammatory responses in the ischemic brain [9, 11, 13, 27].

Peroxiredoxins are a family of oxidative stress-inducible antioxidant enzymes that are especially abundant in brain cells, likely because of the presence of ROS-sensitive unsaturated fatty acids in brain cell membranes [28–30]. Both transgenic and adenovirus-mediated overexpression of intracellular Prxs protects neurons from ischemic and excitotoxic injury by scavenging ROS and inhibiting apoptosis *in vivo* [31, 32]. Extracellular Prxs that are released from ischemic brain cells over 12 h after stroke may act as inflammatory DAMPs, leading to the activation of TLR2/4 on immune cells and inflammatory responses in the brain. Systemic administration of a mixture of antibodies that are specific to Prxs 12 h after cerebral ischemia effectively improved neurological outcomes in MCAO mice [9, 33]. Oxidative stress induces Prx expression and catalyzes cysteine oxidation in Prx1 and Prx2 that inactivates their antioxidative activity and triggers their secretion from cells upon exposure to inflammatory stimuli [11, 27, 34]. Only three subtypes of exogenous recombinant Prxs (Prx1, Prx2, and Prx4) strongly activate the TLR4-mediated NF-κB signaling pathway and inflammatory response in RAW264.7 macrophages [13]. Cerebral postischemic redox imbalance induces the oxidative stress-inducible expression and secretome of Prx1/2/4, which may trigger neuroinflammatory injury via the induction of TLR4 signaling activation and production of proinflammatory mediators in both resident and invading immune cells in the ischemic brain. We hypothesized that the inhibition of Prx1/2/4 signaling through direct blockade of their inflammatory effects or attenuation of their expression and spillage by suppressing oxidative stress may be a potential therapeutic strategy with an extended time window for the treatment of acute ischemic stroke.

Gastrodin has been reported to be a potential neuroprotective agent against cerebral ischemia with antiinflammatory and antioxidative activity both *in vitro* and *in vivo* [17, 35, 36]. We first evaluated the efficacy of Gas and Gas-D *in vitro*. The results showed that Gas-D (20 μM) significantly inhibited the Prx1-, Prx2, and Prx4-induced production of the inflammatory mediators NO and TNFa and TLR4 signaling activation in macrophages. Gas-D (1–10 μM) significantly attenuated H_2_O_2_-induced cytotoxicity and the levels of ROS and 4-HNE in nerve cells. In contrast, Gas was ineffective in both cell models under the same conditions. These results are inconsistent with previous studies that investigated the effects of a higher concentration of Gas (30 μM) on lipopolysaccharide- and amyloid-b(1–42)-induced inflammatory and oxidative responses in cells [35, 36]. Our findings demonstrate the stronger antiinflammatory and antioxidative effects of Gas-D compared with Gas *in vitro*, suggesting that Gas-D exerts neuroprotective effects within an extended time window by blocking the Prx1/2/4-induced neuroinflammatory response and inhibiting the redox-dependent expression and spillage of Prx1/2/4 in acute cerebral ischemia.

Furthermore, we used a rat model of MCAO to investigate the effects of Gas and Gas-D on acute cerebral ischemia. The dosage regimen was based on the reported effective dose of Gas and time profile of the extracellular release of Prxs after MCAO. Administration of Gas at a dose of 100 mg/kg, i.p., at the time of reperfusion after MCAO effectively decreased cerebral ischemic damage and oxidative and inflammatory responses [17]. The extracellular release of Prxs mainly occurs 12–24 h after MCAO [9, 33]. Therefore, we administered 100 mg/kg Gas and Gas-D (i.p.) 10 h after reperfusion following 2-h MCAO and evaluated their effect on brain injury and redox-dependent Prx1/2/4 signaling 22 h after reperfusion. Delayed Gas-D administration significantly ameliorated neurological deficits, cerebral infarction, and neuropathological changes, including a decrease in NeuN-positive neurons and increase in glial fibrillary acidic protein-positive astrocytes, Iba-1-positive microglia/macrophages, and CD3-positive T-lymphocytes in the ischemic cerebral cortex. Gas-D significantly decreased the immunoreactivity of 4-HNE, a typical marker of lipid peroxidation. Moreover, immnunostaining and immunoblotting indicated that Gas-D reduced Prx1/2/4 expression and release from cells into CSF in the ischemic brain. Considering the potent antioxidative effects of Gas-D against H_2_O_2_-induced oxidative stress *in vitro* and in brain ischemia *in vivo*, we hypothesize that Gas-D inhibits redox-dependent Prx1/2/4 signaling and subsequent immunoinflammatory responses after cerebral ischemia by restoring the postischemic redox imbalance. We further examined the effects of Gas-D on TLR4 signaling and downstream response genes in activated immune cells. Immunoblotting and qPCR showed that Gas-D inhibited TLR4 expression, NF-kB activation, and the mRNA levels of the proinflammatory mediators TNF-a, iNOS, MMP-9, and IL-17. These data demonstrate that the neuroprotective effect of Gas-D is associated with the inhibition of TLR4 signaling in acute cerebral ischemia. In contrast to the reported neuroprotective effect of early Gas administration at the time of reperfusion after MCAO [16, 17], delayed Gas treatment did not improve neurological outcomes after ischemic stroke.

Altogether, the present study found that a newly synthesized small-molecule compound, Gas-D, has a wide therapeutic time window of at least 12 h after acute ischemic stroke and exerts its protective effects by inhibiting Prx1/2/4-TLR4 signaling, neuroinflammation, and the redox-dependent expression and secretome of Prx1/2/4. Further pharmacological studies are needed to explore the therapeutic time window of Gas-D administration and evaluate its dose-dependent neuroprotective effects in various models of ischemic stroke.

## MATERIAL AND METHODS

### Reagents

The primary antibodies are summarized in Table 1. The ROS assay kit was obtained from Beyotime (Shanghai, China). Trizol reagent was purchased from Invitrogen Life Technologies (Carlsbad, CA, USA). The DeadEnd Fluorometric TUNEL System was obtained from Precision Design for Life (Beijing, China). The specific primer pairs for polymerase chain reaction (PCR) were obtained from Beijing Genomics Institute (Beijing, China; Table 2). Recombinant mouse Prxs were purchased commercially: Prx1 (batch no. 50552-mM08E; Sino Biological, Beijing, China), Prx2 (batch no. RPF757Mu01, USCN Life Science, Wuhan, Hubei, China), and Prx4 (batch no. RPF754Mu01, USCN Life Science, Wuhan, Hubei, China). The Prxs were prepared as a 10 μM stock solution and kept at -80°C according to the manufacturers’ instructions. The solutions were diluted with cell culture medium before use. All of the other reagents were obtained from local commercial sources.

**Table 1:** Primary antibodies used in this study

Antibody	Dilution	Application	Source
CD3	1:100	IHC^a^	Abcam, USA
4-HNE	1:100	ICC^b^	
Prx1	1:1000; 1:100	WB^c^; IF^d^	
Prx2	1:1000; 1:100	WB; IF	Santa Cruze, USA
Prx4	1:1000; 1:100	WB; IF	
TLR4	1:1000; 1:100	WB; ICC	
iNOS	1:100	ICC	Boster, China
GFAP	1:100	IHC	
Lamin B	1:400	WB	
Neu N	1:100	IHC	Millipore, USA
β-actin	1:1000	WB	
Iba-1	1:500	IHC	Wako, Japan
NF-κB p65	1:400; 1:100	WB; ICC	Zhongshan Jinqiao, China

^a^ IHC, Immunohistochemistry; ^b^ ICC, Immunocytochemistry; ^c^ WB, Western blotting; ^d^IF, Immunofluorescence.

**Table 2:** The specific primer pairs used in polymerase chain reaction

Gene	Forword	Reverse	Anealing temperature (°C)
TNF-α	5′-TCAGCCTCTTCTCATTCCTGC -3′	5′-TTGGTGGTTTGCTACGACGTG -3′	57.4
iNOS	5′-GATATCTTCGGTGCGGTCTT A -3′	5′-GGCCAGATGCTGTAACTCTT -3′	53.1
MMP-9	5′-ACGAGGACTCCCCTCTGCAT -3′	5′-AGGCCTTGGGTCAGGTTTAGA -3′	59.0
IL-17	5′-ACTACCTCAACCGTTCCACTTCA -3′	5′-TGTGCCTCCCAGATCACAGA -3′	56.0
β-actin	5′-AACCCTAAGGCCAACAGTGAAAA -3′	5′-TCATGAGGTAGTCTGTGAGGT -3′	54.0∼59.0

Gastrodin and Gas-D were prepared by Dr. Chu Chen as described previously [20]. Purity (> 98%) was based on the percentage of total peak areas by high-performance liquid chromatography (Supplementary Figure 1). In the present study, Gas and Gas-D were formulated daily in saline with 0.2% dimethylsulfoxide (DMSO) and 3% Tween-80 for the animal experiments or prepared as 50 mM stock solutions in DMSO for the cell experiments, which were diluted with serum-free culture medium with a final concentration of 0.1% DMSO before use.

### Peroxiredoxin-induced inflammatory response in RAW264.7 macrophages

Murine macrophage-like RAW264.7 cells were obtained from KeyGEN Biotechnology (NanJing, Jiangsu, China) and cultured as described previously [13]. The effects of Gas and Gas-D pretreatment for 1 h on the Prx1-, Prx2-, and Prx4-induced inflammatory response were determined by measuring cell viability, inflammatory mediators, and TLR4/nuclear factor kB (NF-kB) signaling in macrophages.

#### Cell viability assay

Cell viability was measured by the MTT assay [37]. The cells (5 × 10^5^ cells/well) were incubated with 20 nM Prx1, Prx2, and Prx4 for 24 h in the presence or absence of Gas or Gas-D at the indicated concentrations. Absorbance was read at 490 nm by a microplate reader (Bio-Rad, Hercules, CA, USA). The data are expressed as a percentage of the vehicle (0.1% DMSO)-treated control group.

#### Determination of inflammatory mediators

To determine the effects of Gas and Gas-D on the production of inflammatory mediators in macrophages, cells (5 × 10^5^ cells/well) were incubated with 20 nM Prx1, Prx2, and Prx4 for 24 h in the presence or absence of Gas or Gas-D at the indicated concentrations. The culture supernatants were then collected to measure nitric oxide (NO) using a NO assay kit (JianCheng Bioengineering Institute, NanJing, Jiangsu, China) and TNF-α using enzyme-linked immunosorbent assay (ELISA) kits (Dakewe Biological Technology, Shenzhen, China) according to the manufacturer’s instructions.

#### Immunocytochemistry and immunofluorescent analysis

Immunocytochemistry was used to detect the expression of iNOS and TLR4 in cells according to the SABC kit procedure. The cells were treated with Prx1, Prx2, and Prx4 for 24 h in the presence or absence of 20 μM Gas or Gas-D and then incubated with a primary antibody (Table 1). Immunoreactivity was visualized using 3,3′-diaminobenzidine tetrahydrochloride (DAB), and photomicrographs were acquired as described previously [13]. To examine the nuclear translocation of NF-κB p65, the cells were incubated with a primary antibody against NF-κB p65 (Table 1), followed by detection with a fluorescein-conjugated secondary antibody (1:100; Boster Biological Technology, Wuhan, Hubei, China). After washing, the nuclei were counterstained with 2-(4-amidinophenyl)-6-indolecarbamidine dihydrochloride (DAPI; Boster Biological Technology). The subcellular localization of NF-κB p65 was observed using a fluorescence microscope (Nikon), and photomicrographs were acquired [13].

### H_2_O_2_-induced oxidative injury in SH-SY5Y cells

Human SH-SY5Y neuroblastoma cells were obtained from the American Type Culture Collection (Manassas, VA, USA) and cultured as described previously [38]. The effects of Gas and Gas-D pretreatment for 24 h on H_2_O_2_-induced oxidative injury were determined by measuring cell viability, ROS, and lipid peroxidation in nerve cells.

#### Cell viability assay

Cell viability was measured using the MTT assay. The cells (1 × 10^4^ cells/well) were incubated with H_2_O_2_ for 24 h in the presence or absence of Gas or Gas-D at the indicated concentrations. Absorbance at 490 nm was measured by a microplate reader (Bio-Rad, Hercules, CA, USA). The data are expressed as a percentage of the vehicle (0.1% DMSO)-treated control group.

#### Determination of ROS generation

The level of intracellular ROS was measured using the ROS assay kit according to the manufacturer’s instructions. Briefly, 24 h after treatment with 10 μM Gas or Gas-D, the cells (1.5 × 10^4^ cells/well) were incubated with fresh serum-free medium that contained 10 μM 2′,7′-dichlorofluorescein diacetate (DCFH-DA) for 30 min at 37°C and then 300 μM H_2_O_2_ for 1 h. Fluorescence was then read at 525 nm with a fluorescence microscope (Nikon, Tokyo, Japan). The generation of ROS was determined by measuring the mean fluorescence intensity of three random fields under a 20´ objective. The data are expressed as a percentage of the vehicle (0.1% DMSO)-treated control group [39].

#### Immunocytochemistry

Immunocytochemistry was used to detect 4-hydroxynonenal (4-HNE) expression in cells according to the SABC kit procedure. The cells were treated with H_2_O_2_ for 3.5 h in the presence or absence of 10 μM Gas or Gas-D and then incubated with a primary antibody against 4-HNE (Table 1). Immunoreactivity was visualized using DAB (Boster Biological Technology) as the chromagen. The expression of 4-HNE was observed under a microscope, and photomicrographs were acquired using a 40× objective and processed using SPOT advanced 4.6 software (SPOT Imaging Solutions, Sterling Heights, MI, USA) [40].

### Cerebral ischemia-reperfusion injury in rats

#### Induction of focal cerebral ischemia and pharmacological treatments

Transient focal ischemia was induced by right MCAO in rats using the intraluminal filament technique as described previously with minor modifications [41, 42]. All of the animal procedures were performed according to China Animal Welfare Legislation and the Guidelines of Laboratory Animal Care and Use of Sichuan University. Specific pathogen-free male Sprague-Dawley rats (310–350 g body weight) were purchased from Chengdu Dossy Experimental Animals Co. Ltd (Chengdu, China). To confirm cerebral ischemia and reperfusion, cerebral blood flow (CBF) in the right middle cerebral artery was monitored from the temporal bone surface at a site 1 mm posterior to bregma and 3 mm inferior to the temporal line using a laser Doppler flowmeter (VMS-LDF, Moore, United Kingdom) [43]. Two hours after MCAO, the thread was carefully withdrawn to establish reperfusion. Rats with both sufficient ischemia (< 20% of baseline) and reperfusion (≤ 80% of baseline) were included in the study. The sham-operated control animals underwent similar surgical procedures without MCAO. Gas or Gas-D (100 mg/kg, i.p.) was administered in MCAO rats 10 h after reperfusion following 2-h MCAO. The sham and MCAO groups received the volume-matched vehicle.

#### Examination of neurological deficits

Neurological impairment after ischemic insult was evaluated using a neurobehavioral test that was scored on a 5-point scale: 0 (no significant deficits), 1 (failure to extend left forepaw fully), 2 (circling to the left), 3 (falling to the left), 4 (inability to walk spontaneously combined with depressed levels of consciousness). Neurobehavioral testing was performed 10 and 22 h after reperfusion following 2-h MCAO by two examiners who were blind to the experimental groups [42].

#### Determination of infarct volume

Twenty-two hours after reperfusion following MCAO, six rats from each group after the neurobehavioral test were euthanized by rapid decapitation. The brains were rapidly removed and frozen at –20°C for 15 min. Coronal brain sections (2 mm thickness) were stained with 0.5% 2,3,5-triphenyltetrazolium chloride (TTC; Sigma-Aldrich, St. Louis, MO, USA). The infarct volume was measured by an investigator who was blind to the treatment groups as described previously [41, 42].

#### Immunohistochemical staining

Twenty-two hours after reperfusion following MCAO, six rats from each group after the neurobehavioral test were deeply anesthetized and perfused through the heart with cold phosphate-buffered saline, followed by 4% paraformaldehyde. Ischemic brain tissue at the level of bregma +0.7 to −2.3 mm in the ipsilateral hemisphere was obtained and used for immunohistochemical staining. Paraffin-embedded brain sections (5 μm) were incubated with appropriately diluted primary antibody (Table 1). Immunoreactivity was visualized using DAB, and counterstaining was performed using hematoxylin. For the semiquantitative analysis of the immunohistochemical results, three sections from each brain, with each section containing three microscopic fields from the ischemic boundary zone (penumbra) in the cerebral cortex, were digitized under a 40´ objective. The immunoreactivity of the target proteins was quantified based on the number of positive cells or integrated optical density (IOD) of immunostaining per field using ImagePro Plus 6.0 software [41, 42].

To determine the extracellular release of Prxs in the ischemic cortex, the brain sections were first stained with the DeadEnd Fluorometric apoptosis detection system (Promega, Madison, WI, USA) for TUNEL staining according to the manufacturer’s instructions, followed by immunohistochemical staining of Prx1, Prx2, and Prx4 as described previously [42]. The localization of Prx1, Prx2, and Prx4 was observed using a fluorescence microscope. Photomicrographs were acquired using a 20´ objective and processed using SPOT advanced 4.6 software (SPOT Imaging Solutions, Sterling Heights, MI, USA).

#### Quantitative real-time polymerase chain reaction

The cerebral cortex of the ipsilateral hemisphere was obtained at 22 h after reperfusion following MCAO. Total RNA was isolated using Trizol reagent and processed for cDNA, followed by quantitative real-time PCR (qPCR) as described previously [42]. The specific primer pairs (Beijing Genomics Institute, Beijing, China) are listed in Table 2. The mRNA levels of inflammatory mediators were normalized to the value of β-actin. The results are expressed as fold changes of the threshold cycle (Ct) value relative to sham-operated controls using the 2^-ΔΔC^_T_ method.

#### Western blot

Twenty-two hours after reperfusion following MCAO, cerebrospinal fluid (CSF) was collected from the cisterna magna for the determination of Prx1, Prx2, and Prx4 content. The cerebral cortex of the ipsilateral hemisphere was obtained 22 h after reperfusion following MCAO, and total protein and nuclear protein were isolated as described previously [41, 42]. Equal amounts of brain tissue protein or 20 µl of CSF were separated by 10% sodium dodecyl sulfate-polyacrylamide gel electrophoresis and transferred to a polyvinylidene fluoride membrane (Millipore, Bedford, MA, USA). The membrane was then incubated with a primary antibody (Table 1) overnight at 4°C, followed by incubation with an appropriate horseradish peroxidase-conjugated secondary antibody (Zhongshan-Golden Bridge, Beijing, China) for 1 h at room temperature and an ECL chemiluminescence kit (Millipore, Billerica, MA, USA). The optical density (OD) of each band was determined using Gel Pro Analyzer 6.0 (Media Cybernetics, Bethesda, MD, USA). The immunoblot results were quantitatively analyzed densitometrically.

### Statistical analysis

The data are expressed as mean ± SEM and were analyzed using one-way analysis of variance (ANOVA) followed by the Tukey test. Values of *p* < 0.05 were considered statistically significant.

## SUPPLEMENTARY MATERIALS FIGURES


