# Supplementary material for: Neuroprotective effect of a novel gastrodin derivative against ischemic brain injury: involvement of peroxiredoxin and TLR4 signaling inhibition
Source: Oncotarget. 2017 Jun 28;8(53):90979–95. doi: 10.18632/oncotarget.18773 (PMC5710899; doi:10.18632/oncotarget.18773)
Supplement: Supplementary file 1 [file oncotarget-08-90979-s001.pdf]

# Neuroprotective effect of a novel gastrodin derivative against ischemic brain injury: involvement of peroxiredoxin and TLR4 signaling inhibition

## Supplementary Materials

**A** Gastrodin (Gas)

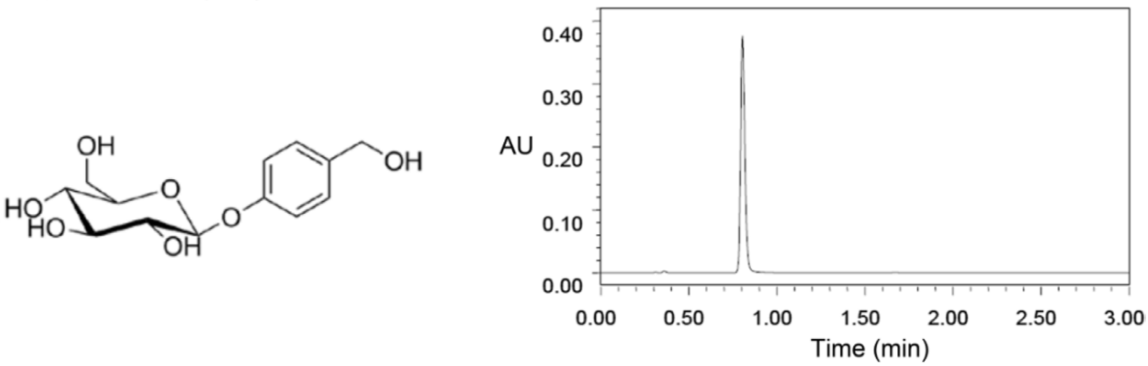


**B** Gastrodin derivative (Gas-D)

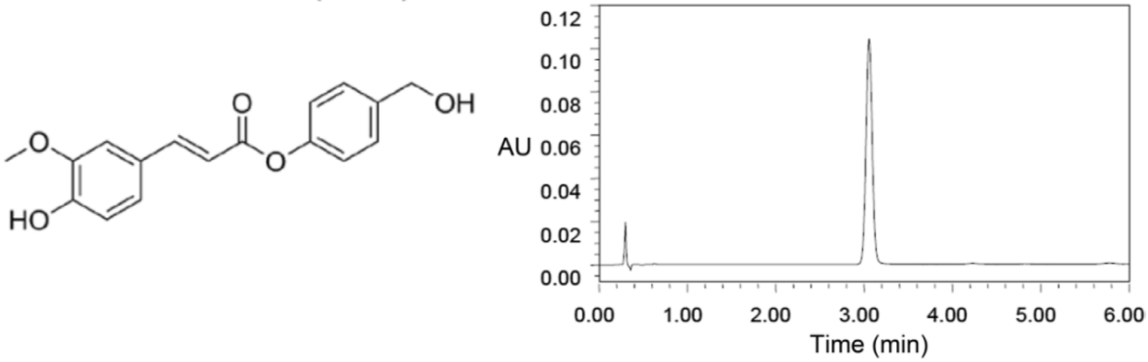


**C** Ferulic acid

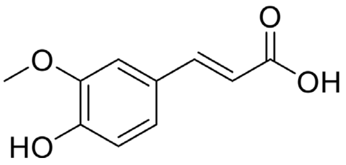


**Supplementary Figure 1: Chemical structures and UPLC chromatograms of compounds.** The separations were performed on a Waters BEH C18 (50 × 2.1 mm i.d., 1.7 µm) column. The mobile phase consisted of acetonitrileand 0.1% aqueous phosphoric acid (v/v, B) using isocratic programs of 3% A for gastrodin (**A**) and 25% A for gastrodin derivative (**B**) respectively. The flow rate was 0.4 ml/min, and the column temperature was maintained at 30°C. The detective wavelengths for Gas and Gas-D were set at 220 nm and 330 nm, respectively. As a result, the respective purities of the tested compounds were more than 98.0% based on peak area normalization. (**C**) Chemical structure of ferulic acid.

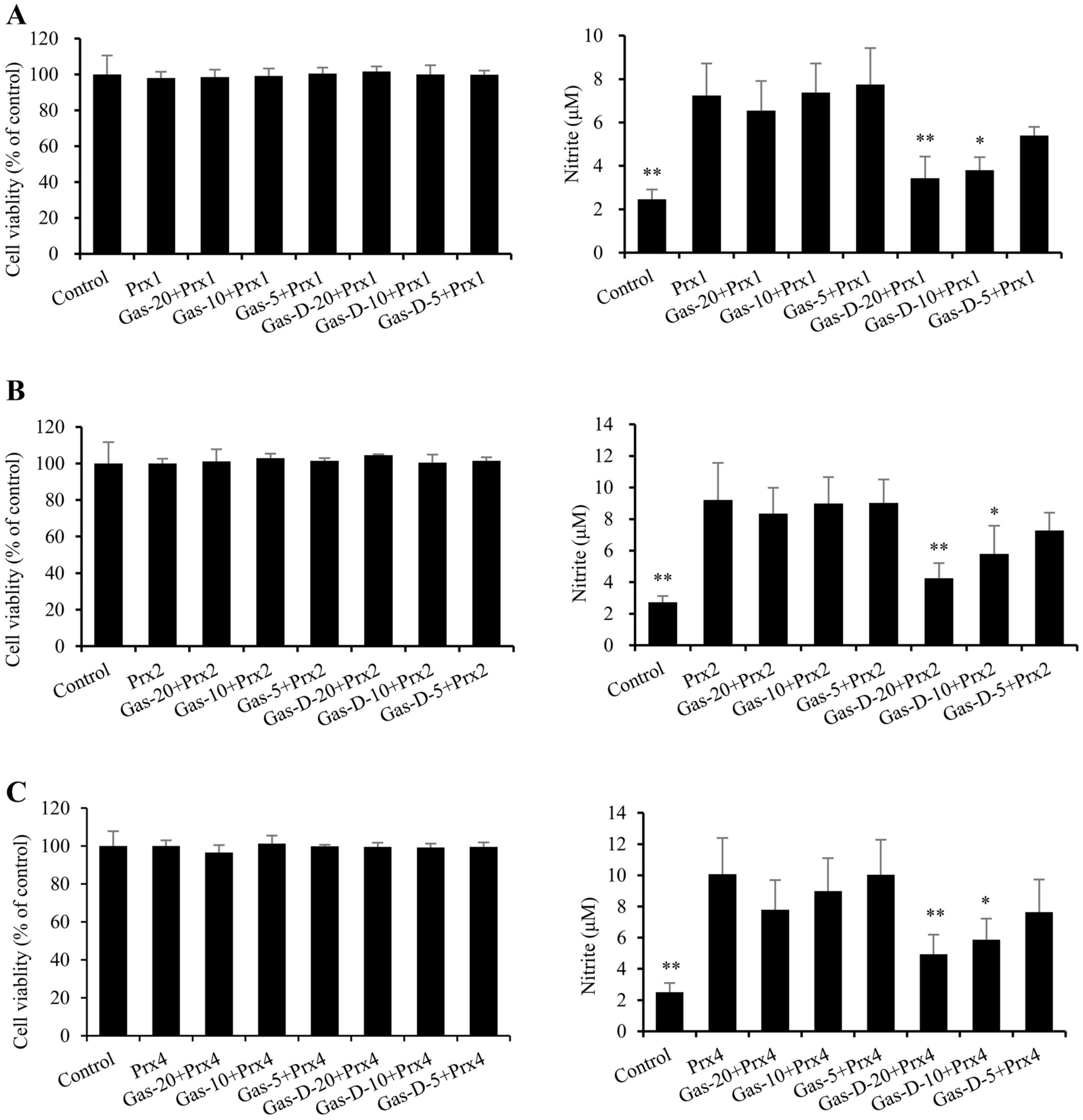


**Supplementary Figure 2: Dose-dependent effects of Gas and Gas-D on cellular viability and NO production in macrophages treated with Prxs.** RAW264.7 cells were pretreated for 1 h with vehicle, Gas, or Gas-D at doses of 5, 10, or 20 μM and then incubated with vehicle (Control) or the indicated Prx subtype (Prx1, Prx2, or Prx4) at a dose of 20 nM for 24 h. Cell viability was examined by the MTT assay, and NO content was measured by a NO kit. (**A–C**) Effects of Gas and Gas-D on Prx1-, Prx2-, and Prx4-stimulated cells. The results are expressed as a percentage of the control group. The data are representative of three independent experiments with 8 or 6 replicates for the MTT and NO assays, respectively.

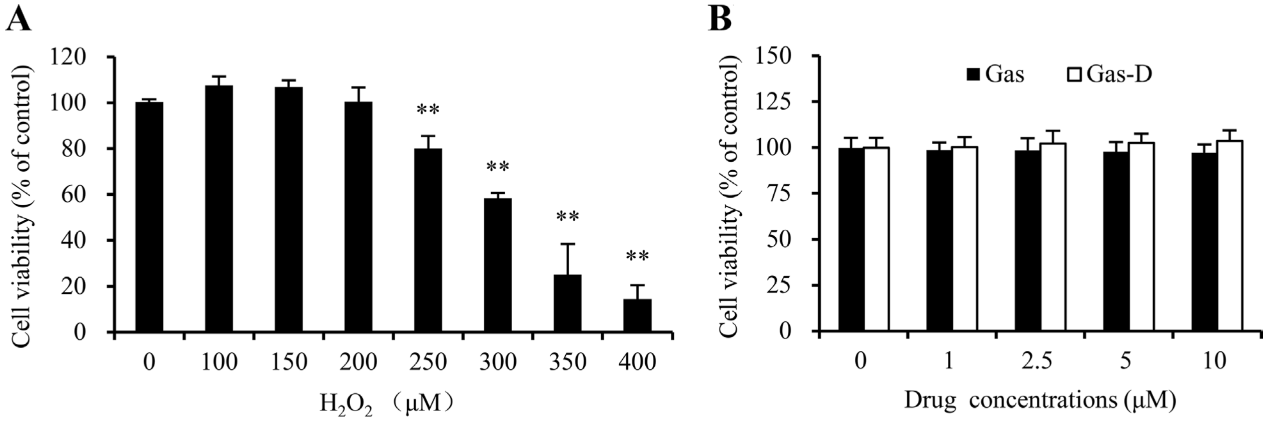


**Supplementary Figure 3: Cytotoxic effects of H2O2, gastrodin (Gas), and its derivative (Gas-D) in SH-SY5Y cells.** Cells were incubated with H2O2 at the indicated concentrations for 24 h, and cell viability was examined by the MTT assay. The results are representative of three independent experiments. The data are expressed as mean ± SEM (*n* = 6). **p* < 0.05, ***p* < 0.01, vs. vehicle-treated control group.
